# Are We Following iRefer Guidelines From the Royal College of Radiology When Requesting Abdominal X-rays?

**DOI:** 10.7759/cureus.31050

**Published:** 2022-11-03

**Authors:** Taimoor Hassan, Mick Kumwenda, Saad Qutab, Saman Z Naqvi, Hamayun Rashid, Sarah Johnson

**Affiliations:** 1 Medicine, Glan Clwyd Hospital, Rhyl, GBR; 2 Nephrology, Glan Clwyd Hospital, Rhyl, GBR; 3 Pulmonology, Glan Clwyd Hospital, Rhyl, GBR; 4 Surgery, Betsi Cadwaladr University Health Board, Rhyl, GBR; 5 Emergency Department, University Hospital Coventry & Warwickshire, Coventry, GBR; 6 Radiology, Betsi Cadwaladr University Health Board, Rhyl, GBR

**Keywords:** acute surgical abdomen, axr, intestinal obstruction, irefer guidelines, abdominal x-rays

## Abstract

Background

Abdominal radiography is one of the most routinely performed radiological investigations in hospitals. It is one of the initial investigations done in hospitals. Numerous studies have shown that abdominal X-rays have low sensitivity in several conditions such as acute abdominal pain.

Methodology

This study aims to first identify whether the Royal College of Radiology guidelines are being adhered to while requesting abdominal X-rays and, second, to identify the number of unnecessary requests made in the Betsi Cadwaladr health board. This is a retrospective audit of abdominal X-ray request data collected between the 1st and 23rd of August 2022. Data were collected from the electronic radiology record system. iRefer guidelines by the Royal College of Radiology were used as a reference to compare the requests made, and data were then analysed accordingly. Data are reported descriptively using percentages. Data analysis was done using SPSS version 20 (IBM Corp., Armonk, NY, USA).

Results

Of the total 242 abdominal X-rays noted, 89.67% of requests were according to the iRefer guidelines while 10.33% of requests were not. A total of 73.14% of cases were suspected to have an intestinal obstruction, and the positivity rate for the same was only 12.39%.

Conclusions

The majority of the requests followed the guidelines. However, there is an urgent need to develop local guidelines to reduce needless abdominal X-rays.

## Introduction

Radiological investigations are often essential for the confirmation of a diagnosis. In 1895, Wilhelm Röntgen made the landmark discovery of electromagnetic radiation with a short wavelength known as X-rays. Initially, X-rays were only used to investigate bone fractures and foreign bodies, but over time they were also applied in the diagnosis of other conditions such as acute abdominal pain. At least 5-10% of visits to the emergency department are for the chief complaint of acute abdominal pain [1]. A plain abdominal X-ray is one of the most common radiological investigations requested by hospital personnel. The utility rate of an abdominal X-ray has been questioned for many of its conditions; radiation exposure is 0.7 millisievert (mSv) with an abdominal X-ray compared to 0.1 mSv with a chest X-ray and 10.0 mSv with computed tomography (CT) of the abdomen [2,3]. Additionally, several studies have shown that CT scan is more beneficial both in early diagnosis and minimizing mortality [4]. In past literature, it has been reported that surgeons often request abdominal X-rays as a part of their routine examination to diagnose undifferentiated abdominal pain. The Royal College of Radiology (RCR) has restricted indications for abdominal X-rays to suspected bowel obstruction, constipation, palpable mass, acute exacerbation of inflammatory bowel disease, foreign body, acute, and chronic pancreatitis, or abdominal injury due to stabbing [5].

## Materials and methods

This is a retrospective audit of abdominal X-rays requested across three hospitals. The participants in this study were patients who were enrolled and had an abdominal X-ray between the 1st and 23rd of August 2022.

All patients above 18 years of age were eligible for the study, while abdominal X-rays done for urological indications and for patients in the pediatric age group (age less than 18 years) were excluded from the study.

We aimed to analyze the indications for abdominal X-rays. Data were collected from the electronic system of the radiology department at Betsi Cadwaladr Health Board. Patients’ demographics were recorded and their clinical details were collected which were then analysed and reported descriptively as percentages. Compliance with known standards from the RCR was checked.

Patients were identified through the results from the radiology department, and details on request forms and radiological reports were analyzed to check whether abdominal X-rays were indicated in line with RCR guidelines (Table 1) and whether any positive radiological findings were further confirmed by CT imaging. The X-rays showing positive findings were correlated with the signs and symptoms of the patients.

**Table 1:** RCR iRefer guidelines for plain abdominal radiography [5]. RCR: Royal College of Radiology

RCR iRefer guidelines for plain abdominal radiography
Clinical suspicion of obstruction
Acute exacerbation of inflammatory bowel disease
Palpable mass (specific circumstances)
Constipation (specific circumstances)
Acute and chronic pancreatitis (specific circumstances)
Sharp/poisonous foreign body
Smooth and small foreign body, e.g., coin, battery (specific circumstances)
Blunt or stab abdominal injury (specific circumstances)
Post-Gastrografin follow-through study

## Results

A total of 242 abdominal films were identified. A total of 89.67% of abdominal X-rays were requested according to the iRefer guidelines while 25 abdominal X-rays were done, i.e., 10.33% of all requests, against the iRefer guidelines of RCR. A greater majority of the population (73.14%) was investigated for abdominal obstruction, accounting for a total of 177 patients. In this 73.14%, the top clinical detail of requesting an abdominal X-ray with suspicion of abdominal obstruction was abdominal pain, accounting for 19.20%. Likewise, abdominal pain with the bowel not opened and abdominal pain with vomiting accounted for 11.30% and 9.60%, respectively. Abdominal pain with each loose stool, distension, and distension plus vomiting accounted for 2.26%, 11.30%, and 3.95%, respectively. A total of 15.82% of requests had clinical details of abdominal distension alone and with vomiting, and with the bowel not opened. Similarly, vomiting, loose stools, reduced bowel sounds, and bowel not opened requests made up a total of 16.93% of the inquiries of abdominal obstruction. While the rest of the requests together made up 9.58% (Table 2).

**Table 2:** Frequency and percentages of the various clinical details provided when abdominal obstruction X-rays were ordered.

Clinical details of abdominal obstruction requests	Total (n)	Percentage (%)
Abdominal pain	34	19.20
Abdominal pain, bowel not opened	20	11.30
Abdominal pain, vomiting	17	9.60
Abdominal pain, loose stool	4	2.26
Abdominal pain, distension	20	11.30
Abdominal pain, bowel not opened, diarrhoea	1	0.56
Abdominal pain, bowel not opened, vomiting	9	5.08
Abdominal pain, vomiting, constipation, distension	1	0.56
Abdominal pain, vomiting, diarrhoea	1	0.56
Abdominal pain, distension, vomiting	7	3.95
Abdominal pain, bloody stools	3	1.69
Abdominal distension	16	9.04
Abdominal distension, bowel not opened	8	4.52
Abdominal distension, bowel not opened, vomiting	2	1.13
Abdominal distension, vomiting	4	2.26
Vomiting	8	4.52
Vomiting, loose stools	1	0.56
Vomiting, bowels not opened	9	5.08
Bowels not opened	11	6.21
Reduced bowel sounds	1	0.56
Total	177	

A total of 2.47% of inquiries were about toxic megacolon in acute exacerbation of inflammatory bowel disease. Likewise, 5.37% of requests were to check for foreign bodies and observe the position of the percutaneous endoscopic jejunostomy (PEJ) tube, peritoneal dialysis catheter position nasojejunal (NJ) tube position, etc. While 2.47% of abdominal X-rays were requested to see toxic megacolon in *Clostridium difficile*. One X-ray was requested to check for a palpable abdominal mass. Similarly, four abdominal X-rays were done for the post-Gastrograffin study, and eight were done to observe faecal loading to assess constipation. The patients with positive findings of bowel obstruction on abdominal X-rays were further investigated by CT scans based on the clinical assessment. Based on the finding of the abdominal film, a CT abdomen was requested on 21 patients. A total of 15 of these CT scans confirmed the suspicion raised on the plain abdominal film while six of the CT Scans were reported normal (Table 3).

**Table 3:** Indications for initial abdominal X-ray, positive findings thereafter, and any further CT done or CT confirming a diagnosis and where no further investigation was done are presented as frequencies. CT: computed tomography

Indications	Number of patients (n)	Positive finding	Further CT done	CT confirmed diagnosis	No further investigation done
Bowel obstruction	177 (73.14%)	30 (12.39%)	21 (8.67%)	15 (6.19%)	9 (3.71%)
Exacerbation of inflammatory bowel disease	8 (3.30%)	0			
Foreign body	13 (5.37%)	3 (1.20%)			
*C. difficile* toxic megacolon	6 (2.47%)	0			
Post-Gastrografin study	4 (1.65%)	0			
Constipation	8 (3.30%)	1 (0.40%)			
Not following iRefer protocol	25 (10.33%)				
Abdominal mass	1 (0.41%)				
Total	242				

## Discussion

Abdominal X-ray is frequently ordered as a part of the investigation for abdominal conditions in hospitals. An abdominal X-ray is the start of the imaging workup [6]. Most patients who have significant findings in abdominal X-rays are referred for further investigations. To reduce inappropriate referrals iRefer guidelines were introduced which enlist recommended indications for abdominal X-rays.

Abdominal X-rays show a sensitivity of 90% in observing intra-abdominal foreign bodies with a sensitivity of 49% in detecting bowel obstruction [7]. Around 242 abdominal X-rays were requested during the 23 days of our study. A total of 177 patients’ abdominal X-ray requests had a clinical suspicion of abdominal obstruction. The variables that were put in the clinical history of abdominal X-ray request forms were mostly abdominal pain, constipation, abdominal distention, and vomiting. The abdominal X-ray reports were positive in only 30 (12.39%) cases. In total, 21 (8.67%) cases were further confirmed by CT scan, of which a minority (6.19%) of CT scan results were consistent with X-ray reports. Acute exacerbation of inflammatory bowel disease is also an indication of abdominal X-rays. In our study, eight patients were assessed by abdominal X-rays for the same. Most requests were for investigating the dilatation of the bowel. Suspicion of constipation accounted for only eight patients in our study population.

Another indication recommended by iRefer for abdominal X-ray is a foreign body. Abdominal X-rays are used to find the position of hazardous bodies, such as sharp objects, and non-metallic objects, such as glass beads and batteries [8]. Plain X-rays show a specificity of 100% and 90% specificity for ingested foreign bodies, respectively, and it has to be radio-opaque [9]. A total of 13 X-rays were requested to investigate foreign bodies in our study which also included the requests to see the PEJ tube, NG tube, and peritoneal dialysis catheter position. A post-Gastrografin follow-through study was done to differentiate between partial and complete small bowel obstruction [10]. Post-Gastrograffin abdominal X-ray is also used commonly, which in our case accounted for four requests in 23 days. Similarly, palpable mass, acute and chronic pancreatitis, and blunt or stabbing abdominal injury are also indications of abdominal X-rays given by iRefer. In our study, we find a greater proportion of clinicians following iRefer guidelines. A prospective observational study of abdominal X-rays done by Feyler et al. showed that from a total of 1,309 patients, 131 had abdominal X-rays done, of which only 12% of the requests were compliant with RCR guidelines [11]. Contrary to that, in our study, the compliance rate with iRefer guidelines was 89.67%. This discrepancy warrants further study, preferably prospective, to determine the reasons. However, the leniency of acceptability of clinical information provided against RCR guidelines is a possible main reason for this huge difference.

Although the referrals for abdominal X-rays are largely in accordance with the iRefer guidelines, the findings of abdominal X-rays suggest that clinical signs and symptoms have limited predictive value for abdominal X-ray abnormalities. The majority of abdominal X-rays had normal results in our study. Similar findings were seen in previous studies where only 15.8% and 25% showed positive X-ray findings [12,13]. The request process for abdominal X-rays has been challenged by several authors [14,15]. There is room for further research and guidelines that need to be refined which might help in reducing unnecessary abdominal X-ray requests.

## Conclusions

This study compared abdominal X-ray requests referring to the iRefer guidelines by the RCR and found that a greater number of abdominal X-rays were requested in concordance with iRefer guidelines. Moreover, only a minimum number of abdominal X-rays had positive findings. Further studies are recommended for the best utilization of abdominal X-rays or for investigating other alternative imaging techniques such as an ultrasound of the abdomen which might help in avoiding unnecessary radiation exposure.

## References

[1] Laméris W, van Randen A, van Es HW (2009). Imaging strategies for detection of urgent conditions in patients with acute abdominal pain: diagnostic accuracy study. BMJ.

[2] Bertin CL, Ponthus S, Vivekanantham H, Poletti PA, Kherad O, Rutschmann OT (2019). Overuse of plain abdominal radiography in emergency departments: a retrospective cohort study. BMC Health Serv Res.

[3] Wall BF, Hart D (1997). Revised radiation doses for typical X-ray examinations. Report on a recent review of doses to patients from medical X-ray examinations in the UK by NRPB. National Radiological Protection Board. Br J Radiol.

[4] Ng CS, Watson CJ, Palmer CR (2002). Evaluation of early abdominopelvic computed tomography in patients with acute abdominal pain of unknown cause: prospective randomised study. BMJ.

[5] Kyriakides J, Khamar R, Khani A, Khatkar H (2022). A quality improvement project: reducing the number of unnecessary plain abdominal radiographs performed in the emergency department of a London district general hospital. J Family Med Prim Care.

[6] Karkhanis S, Medcalf J (2009). Plain abdomen radiographs: the right view?. Eur J Emerg Med.

[7] Ahn SH, Mayo-Smith WW, Murphy BL, Reinert SE, Cronan JJ (2002). Acute nontraumatic abdominal pain in adult patients: abdominal radiography compared with CT evaluation. Radiology.

[8] Smith JE, Hall EJ (2009). The use of plain abdominal x rays in the emergency department. Emerg Med J.

[9] Gans SL, Stoker J, Boermeester MA (2012). Plain abdominal radiography in acute abdominal pain; past, present, and future. Int J Gen Med.

[10] Biondo S, Parés D, Mora L, Martí Ragué J, Kreisler E, Jaurrieta E (2003). Randomized clinical study of Gastrografin administration in patients with adhesive small bowel obstruction. Br J Surg.

[11] Feyler S, Williamson V, King D (2002). Plain abdominal radiographs in acute medical emergencies: an abused investigation?. Postgrad Med J.

[12] Böhner H, Yang Q, Franke C, Verreet PR, Ohmann C (1998). Simple data from history and physical examination help to exclude bowel obstruction and to avoid radiographic studies in patients with acute abdominal pain. Eur J Surg.

[13] Campbell JP, Gunn AA (1988). Plain abdominal radiographs and acute abdominal pain. Br J Surg.

[14] Stower MJ, Amar SS, Mikulin T, Kean DM, Hardcastle JD (1985). Evaluation of the plain abdominal X-ray in the acute abdomen. J R Soc Med.

[15] Field S, Guy PJ, Upsdell SM, Scourfield AE (1985). The erect abdominal radiograph in the acute abdomen: should its routine use be abandoned?. Br Med J (Clin Res Ed).

